# Quantitative genetic analysis of late spring mortality in triploid *Crassostrea virginica*

**DOI:** 10.1186/s12711-025-00965-3

**Published:** 2025-04-09

**Authors:** Joseph L. Matt, Jessica Moss Small, Peter D. Kube, Standish K. Allen

**Affiliations:** 1 Marine Genomics Lab, Department of Life Sciences, Texas A&M University-Corpus Christi, 6300 Ocean Drive, Corpus Christi, TX 78412 USA; 2 Texas A&M AgriLife Research, 600 John Kimbrough Boulevard, Suite 512, College Station, TX 77843 USA; 3 Aquaculture Genetics and Breeding Technology Center, Virginia Institute of Marine Science, William & Mary, P.O. Box 1346, Gloucester Point, VA 23062 USA; 4 Center for Aquaculture Technologies, Hobart, Tasmania Australia; 5 CSIRO Agriculture & Food, Hobart, Tasmania Australia

## Abstract

**Background:**

Triploid oysters, bred by crossing tetraploid and diploid oysters, are common worldwide in commercial oyster aquaculture and make up much of the hatchery-produced *Crassostrea virginica* farmed in the mid-Atlantic and southeast of the United States. Breeding diploid and tetraploid animals for genetic improvement of triploid progeny is unique to oysters and can proceed via several possible breeding strategies. Triploid oysters, along with their diploid or tetraploid relatives, have yet been subject to quantitative genetic analyses that could inform a breeding strategy of triploid improvement. The importance of quantitative genetic analyses involving triploid *C. virginica* has been emphasized by the occurrence of mortality events of near-market sized triploids in late spring.

**Methods:**

Genetic parameters for survival and weight of triploid and tetraploid *C. virginica* were estimated from twenty paternal half-sib triploid families and thirty-nine full-sib tetraploid families reared at three sites in the Chesapeake Bay (USA). Traits were analyzed using linear mixed models in ASReml-R. Genetic relationship matrices appropriate for pedigrees with triploid and tetraploid animals were produced using the polyAinv package in R.

**Results:**

A mortality event in triploids occurred at one site located on the bayside of the Eastern Shore of Virginia. Between early May and early July, three triploid families had survival of less than 0.70, while most had survival greater than 0.90. The heritability for survival during this period in triploids at this affected site was 0.57 ± 0.23. Triploid survival at the affected site was adversely related to triploid survival at the low salinity site (− 0.50 ± 0.23) and unrelated to tetraploid survival at the site with similar salinity (0.05 ± 0.39).

**Conclusions:**

Survival during a late spring mortality event in triploids had a substantial additive genetic basis, suggesting selective breeding of tetraploids can reduce triploid mortalities. Genetic correlations revealed evidence of genotype by environment interactions for triploid survival and weak genetic correlations between survival of tetraploids and triploids. A selective breeding strategy with phenotyping of tetraploid and triploid half-sibs is recommended for genetic improvement of triploid oysters.

**Supplementary Information:**

The online version contains supplementary material available at 10.1186/s12711-025-00965-3.

## Background

Polyploidy has been induced in fish and shellfish species for research purposes and commercial evaluation [1, 2], often with the goal of producing sterile triploid animals. Benefits of sterility for cultured species include improvement in commercial traits, such as more consistent flesh quality [3], as well as reducing risk of genetic exchanges between farmed and wild populations [4]. Triploidy can cause sterility through meiotic dysfunction, largely by the odd number of chromosome sets, but also perhaps from changes in cellular dimensions [5]. Many species of fish and shellfish have been induced to be triploid with varying effects on sterility, from partial to total. In a handful of animal species, triploidy has become commercially useful, such as in some salmonids (*Oncorhynchus mykiss, Salmo salar*) and in oysters (*Crassostrea spp*.) [6, 7].

The commercial potential of triploid oysters was first examined via chemical induction. Triploid oysters were induced by treating fertilized eggs with the fungal antibiotic cytochalasin B [8–10], which produces triploidy by blocking the extrusion of a polar body during meiosis. Such “chemical triploids” exhibited lower fecundity than diploids [11–13] and manifested consistent meat quality during the warmer months, when quality of diploid oysters decreases substantially as a result of spawning [3, 14]. Although commercial production of chemical triploid oysters began in the United States Pacific Northwest of the USA in the late 1980s [3], limitations in large scale production of triploids, including the risk of producing a mix of triploids and diploids from the treatment [8], made desirable a method that would more consistently produce populations that were 100% triploid.

Reliable production of 100% triploid populations became possible with the creation of tetraploid oysters. Experience in plants had demonstrated the value of tetraploids to create triploid progeny when crossed with diploids [15]. Guo and Allen [16] first produced tetraploid oysters by chemical induction, after which Guo et al. [17] demonstrated 100% triploid progeny from crossing tetraploids to diploids. Like chemically induced triploids, these “mated triploids,” have reduced fecundity [18–20] and maintain meat quality during the spawning season [14, 21]. Crossing tetraploids and diploids is now a widely applied method of commercial production of triploid oysters in many parts of the world, including Western Europe, Australia, Mexico, China, as well as the West Coast, Gulf Coast, and East Coast of the United States.

Mated triploid oysters are especially popular in the lower Chesapeake Bay of the United States, a region where hatchery-based oyster aquaculture has grown dramatically over the last 15 years. From 2005 to 2018, the number of market sized *Crassostrea virginica* sold by hatchery-based production in Virginia has increased from less than 5 million to over 30 million [22], and triploids have comprised nearly all production. Since surveys were initiated in 2009, triploids have constituted 80 to 97% of hatchery-produced oysters planted on Virginia farms [22, 23].

Commercial aquaculture of *C. virginica* in Chesapeake Bay has been shaped by both polyploidy and selective breeding. The Aquaculture Genetics and Breeding Technology Center (ABC) at the Virginia Institute of Marine Science (VIMS) has been supplying commercial hatcheries selectively bred diploid and tetraploid brood stock (for diploid × tetraploid crosses) since 2004. Originally, ABC employed mass selection on diploids for faster growth and resistance to diseases caused by the protozoans *Haplospiridium nelsoni* and *Perkinsus marinus* [24, 25]. In 2015, ABC operationalized a pedigree-based family breeding program with diploid oysters. This program has yielded genetic gains in commercial traits, as well as estimates of genetic parameters that have shaped the strategy for selecting diploids suited for high and low salinity zones [26]. Starting in 2018, this same approach was applied to tetraploids in a parallel family breeding program.

The production of triploids and the possible breeding schemes for their improvement are analogous to crossbreeding in crops and livestock. In livestock, individuals of distinct groups are crossed to generate heterosis or to produce offspring from breeds with complementary traits. Although the production of triploid oysters differs in that its primary purpose is for sterility, various breeding strategies for crossbred improvement (review by [27]) can be applied to triploid oysters. Like crossbreds, triploids may be improved by selecting based on the performance of their progenitors (diploids and tetraploids), performance of the triploids themselves (e.g., reciprocal recurrent selection, RRS [28]), or some combination of both (e.g., [29, 30]).

Originally, ABC ran parallel programs for diploid and tetraploid oysters, under the assumption that the additive genetic gains made in both family breeding programs would result in genetic improvement of the mated triploids. However, critical determinants of the success of this strategy, i.e. the genetic correlations between traits in triploids with those in diploids and tetraploids, had not been estimated. In fact, despite the widespread use of mated triploids as a commercial product, commercial traits in triploid oysters had not been subject to quantitative genetic analyses until ABC started to produce related triploid and tetraploid families in 2017.

Late spring mortality events on commercial farms have accentuated the importance of quantitative genetic analysis of traits in triploid oysters. Since 2012, unusual mortality of near-market sized (76 mm) triploids in late spring has been reported at some oyster farms in the Chesapeake Bay and has been the subject of several empirical studies [18, 21, 31]. A typical late spring mortality event involves mortality exceeding 20% of the stock but has approached much higher levels (50 to 85%) in some years. The mortality events are unusual because they are not associated with typical stressors for *C. virginica*, such as regional pathogens, poor husbandry, extreme environmental conditions, or major changes in salinity [18, 21]. The original name, “triploid mortality” [18, 21], represents an early hypothesis that the mortality events were tied to the triploid condition. Although there is some evidence that triploids are especially susceptible [21], some studies have found diploids can die in similar fashion [18, 31].

A recent study by Ritter [31] suggested that selective breeding can be used to address late spring mortality events. In their study, triploid half-sib families that varied based on tetraploid sire were deployed to a site where late spring mortality events regularly take place. Following a late spring mortality event, substantial variance in survival was observed among triploid families. Although heritability for survival was not estimated, this suggested that survival was influenced by genetic effects from the tetraploid sires, implying that selective breeding for resistance to triploid mortality could be effective.

Estimating genetic parameters in polyploid populations, such as heritability and genetic correlations, requires different statistical methods than those that are used for diploid populations. Genetic parameters in animals are traditionally estimated using a mixed linear model with phenotypes and genetic relationships among the observed individuals as inputs. The standard methods of estimating genetic relationships from a pedigree assume all individuals are diploid [32]. When estimating genetic parameters in pedigrees with individuals of multiple ploidy levels, the standard methods provide inaccurate estimates because the number of sets of chromosomes inherited from sire and dam can be disproportionate and, thus, genetic relationships among individuals can be different than in a diploid population. To efficiently determine genetic relationships in mixed-ploidy pedigrees, Hamilton and Kerr [33] established a set of rules and an associated R package, polyAinv.

The primary objective in this study was to estimate the additive genetic basis of late spring mortality events in triploid *C. virginica* to evaluate the potential of selective breeding as a method to reduce mortalities. Additional goals were to estimate genetic correlations of survival between triploids at different sites, between triploids and tetraploids, and between weight and survival in triploids to inform selective breeding strategies for triploid oysters. Quantitative genetic analysis was based on field tests of triploid and tetraploid families conducted from the summer of 2018 to the fall of 2019. To estimate genetic parameters of late spring mortality, 20 triploid families were deployed to a site with a history of triploid mortality events. The same 20 triploid families, as well as 39 tetraploid families that were related to the triploids via the tetraploid sire, were deployed to two additional sites that did not have a history of mortality events. Diploid and tetraploid lines were also deployed to all sites as reference groups. Survival and weight were monitored in families at all sites, and genetic parameters were estimated in triploid and tetraploid families using univariate and multivariate animal models.

## Methods

### Broodstock and crosses

#### Families

Triploid and tetraploid families were produced by crossing individuals from diploid and tetraploid lines. Diploid broodstock consisted of oysters from the ABC line DEBY [25] that had been selected in a low salinity environment (≈ 8–12 ppt), named DEBY LEW, or selected in a higher salinity environment (≈ 18–22 ppt), named DEBY LYN. Tetraploid broodstock were from five ABC lines: 4GEN, 4VBOY, 4OBOY, 4LGT, and 4GNL. The 4GEN line originated from ABC diploid lines and germplasm from Louisiana and has been propagated by ABC since 2003. The 4VBOY, 4OBOY, and 4LGT lines originated as triploids made by crossing diploid lines from Louisiana with the 4GEN line [34]. The 4GNL line is an F1 hybrid of 4GEN and 4VBOY.

A total of 46 tetraploid families and 23 triploid half-sib families were spawned for this study on two dates: June 14, 2017 (24 tetraploid, 12 triploid) and July 10, 2017 (22 tetraploid, 11 triploid) at the VIMS Kauffman Aquaculture Center (KAC) in Topping, Virginia. All crosses were conducted via strip spawning [35]. Full-sib tetraploid families were made by crossing one male and one female. Triploid half-sib families were produced by crossing one tetraploid male with eggs pooled from five diploid females.

Sperm from each tetraploid male was used to make both tetraploid and triploid families. Sperm was split into three aliquots to fertilize eggs from two tetraploid females and a fraction of the eggs from the pooled diploids, producing two full-sib tetraploid families and one paternal half-sib triploid family. For the first spawn, eggs from five females from the diploid line DEBY LEW were pooled, split into 12 aliquots, and fertilized with sperm from 12 tetraploid males. For the second spawn, eggs from five females from the diploid line DEBY LYN were pooled, split into 11 aliquots, and fertilized with sperm from another 11 tetraploid males.

#### Reference lines

Diploid and tetraploids propagated by ABC in 2017 were used as reference lines. Reference diploid lines were included in the experiment to examine the susceptibility of diploids to the mortality episodes in late spring in the lower Chesapeake Bay. Reference tetraploid lines were included so survival could be compared among all ploidies at each site. The pedigree of individuals in reference lines was unknown because each reference line was either produced as a series of crosses among many parents reared as a single batch, or by pooling oysters from several ABC families.

Diploid reference individuals were primarily from lines that are annually propagated by ABC (e.g. [24]): DEBY LYN (herein, DEBY), XB, HNRY, LOLA, and LILY. An additional reference line was a mix of ABC families made in 2017 and labelled LFAMS. DEBY, XB, and HNRY have been selected in higher salinity environments (≈ 18–22 ppt) and are herein referred to as “high salinity” reference lines. LOLA, LILY, and the population sampled to make LFAMS have been selected in lower salinity (≈ 8–12 ppt) and are herein referred to as “low salinity” reference lines.

Three tetraploid reference lines consisted of individuals from the ABC tetraploid lines 4GEN, 4GNL, and 4VBOY, and one was a mix of several VIMS tetraploid families that were created in 2017. The mixed families were spawned from individuals of the ABC tetraploid lines 4OBOY and 4LGT, and therefore the reference line was labelled 4OBLT. All reference lines were spawned at the ABC research hatchery in Gloucester Point, VA, or at KAC. An additional file lists the reference lines by ploidy and environment in which they have been selected (see Additional file 1: Table S1).

### Larval rearing, initial field deployment, and ploidy verification

The triploid and tetraploid families were reared separately in individual tanks at KAC. Larvae were reared at ~ 25 °C, water was changed every two days, and larvae were fed a diet of live *Pavlova pinguis*, *Chaetocerous neogracile*, and *Tetraselmis chuii* based on their development stage. Larvae were harvested once they reached the pediveliger stage (e.g., [36]). Harvesting consisted of sieving the mature pediveligers and storing them in a moist coffee filter at 5 °C. Thereafter, pediveligers were harvested every two days for a total of three harvests. All three harvests for each family were combined and a maximum of 60,000 pediveligers were transferred to individual downweller systems containing oyster shell ground to ~ 400 µm in diameter. When oysters could be retained on a 500 µm screen, families were transferred to individual silos in a land-based upweller system at the ABC research hatchery in Gloucester Point, where they were reared on unfiltered water from the York River.

Once most individuals within a family were larger than 5 mm, each family was deployed into 3 mm mesh bags contained in double-tier bottom cages in the York River (Gloucester Point, VA) at a maximum density of 4,000 oysters per bag. The number of bags deployed per family ranged from one to three, and the time of deployment for each family ranged from August 28, 2017, to November 16, 2017.

Larval husbandry of the reference lines was similar to that of the tetraploid and triploid families. Diploid reference lines were reared at the ABC Gloucester Point hatchery, whereas tetraploid reference lines were reared at KAC. All were transferred to the land-based upweller system at the ABC research hatchery and reared there until large enough to be deployed in bags in the York River in the summer or fall of 2017.

All polyploid families and reference lines were sampled to verify ploidy with a Sysmex-Partex Cyflow Space flow cytometer (Partec GmbH, Münster, Germany), using DAPI as a stain [37]. Fifteen to twenty-five individuals (1–2 mm shell length) were randomly selected from each family or cross while in the upwelling system and were analyzed individually by disaggregating cells from whole individuals. Samples were also taken from 4GEN, 4GNL, and 4VBOY for ploidy verification. The families that made up 4OBLT were verified individually prior to being combined as 4OBLT.

### Field test

**Fig. 1:**
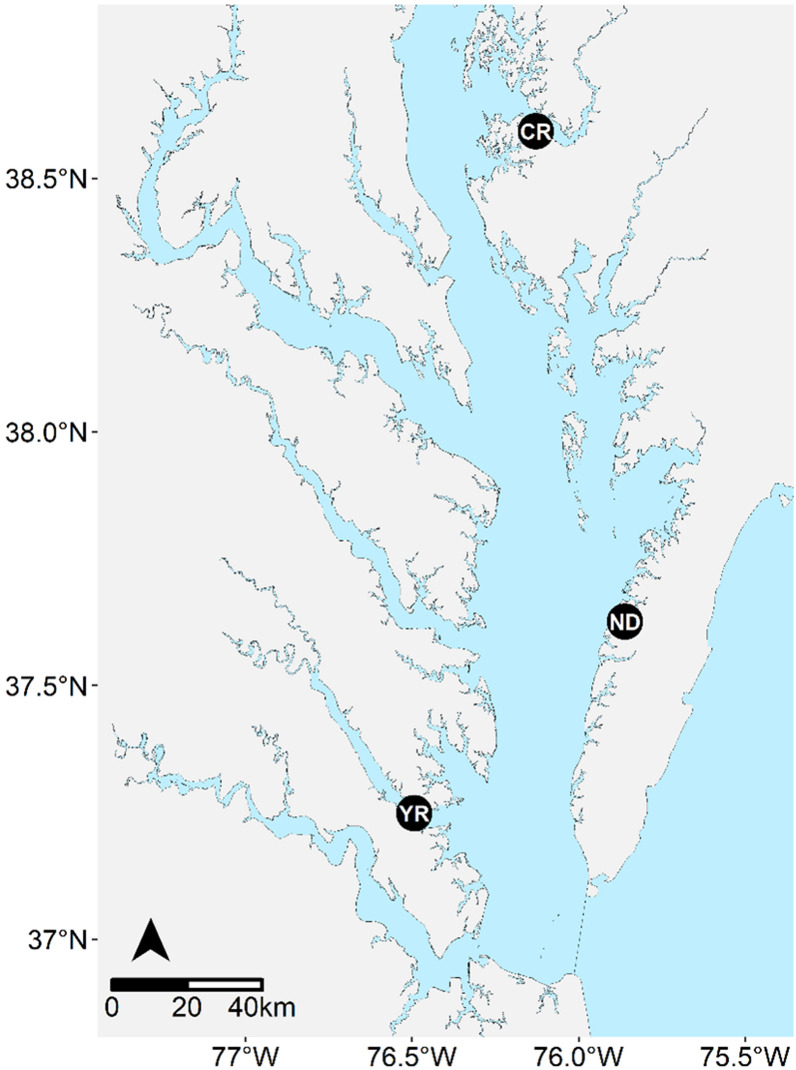
Map of sites in the Chesapeake Bay. Map of where triploid and tetraploid families of *Crassostrea virginica* were deployed for field testing. YR = York River, CR = Choptank River, ND = Nandua Creek

Families and reference lines were deployed for a controlled field test from summer of 2018 to the fall of 2019 at three sites in the Chesapeake Bay: York River (Gloucester Point, VA), Choptank River (Cambridge, MD), and Nandua Creek (Pungoteague, VA) (Fig. 1). Every triploid family was deployed to each site, while tetraploid families were only deployed to York River and Choptank River due to logistical constraints. Diploid high salinity reference lines (DEBY, XB, HNRY) were only deployed to the high salinity sites, York River and Nandua Creek, whereas diploid low salinity reference lines (LOLA, LILY, LFAMS) were deployed to the low salinity site, the Choptank River. An additional file lists the families, reference lines, and their deployment locations (see Additional file 2: Table S2). At the York River and Choptank River, oysters were deployed in baskets on an adjustable longline system. At Nandua Creek, oysters were deployed in vexar bags in single-tier bottom cages.

Individuals deployed to sites for the field test were randomly sampled from each family or reference line on June 4th and 5th of 2018. Each triploid family, diploid reference line, and tetraploid reference line was deployed in three units (bag or basket) of 120 oysters per site, except for 4VBOY, which was deployed in two units of 60 because of low numbers. Each tetraploid family was deployed in three units of 150 oysters to York River and Choptank River, except for six families that were only deployed to York River because of low numbers of individuals within these families. Of the latter six families, 5 were deployed to York River and to Choptank River in two units of 150, and one family was deployed in two units of 120 per site. For all stocking densities (60, 120, 150), oysters occupied less than half their container for the duration of the field test.

Oysters deployed in baskets were removed from the adjustable longline system for winter to avoid exposing oysters to freezing conditions. In December of 2018, baskets at the York River and the Choptank River were removed from the longline system and moved into PVC “Taylor” floats for overwintering. Baskets were returned to the adjustable longline system in March of 2019.

### Environmental conditions

Water temperature was measured continuously at each site during the field trial with HOBO^®^ TidbiT temperature loggers (Onset Computer Corporation, Bourne, MA, USA), and salinity was measured continuously at Nandua Creek during the field trial with a HOBO^®^ conductivity logger. Temperature loggers were placed within a unit (bag or basket) at each site in spring of 2019, while the conductivity logger was placed inside one of the bottom cages at Nandua Creek in winter of 2018. Conductivity was recorded during each site visit to Nandua Creek with a portable conductivity meter (YSI Pro 2030) and was used to calibrate the conductivity data collected by the HOBO logger at Nandua Creek. The conductivity values recorded by the HOBO logger were converted to salinity in parts per thousand (ppt) using the HOBOware Conductivity Assistant. Salinity data for the York River site were accessed via the Virginia Estuarine and Coastal Observing System’s (VECOS, collected and collated by the Chesapeake Bay National Estuarine Research Reserve—Virginia) continuous monitoring station in Gloucester Point, Virginia (station ID: York RiverK005.40). The continuous monitoring station in Gloucester Point collected data with a YSI 6600 data sonde and was approximately 1000 m from the York River site. Salinity data for the Choptank River site was provided by staff at the Horn Point Laboratory at the University of Maryland in Cambridge, Maryland, and was recorded with a portable conductivity meter within 100 m of the test site.

### Survival

Survival was assessed for each unit (bag or basket) in the fall of 2018 (490–546 days old; York River: Nov. 26, Choptank River: Nov. 12, Nandua Creek: Dec. 12), spring of 2019 (658–693 days old; York River: April 29, Choptank River: May 6, Nandua Creek: May 7), summer of 2019 (722–756 days old; York River: July 1, Choptank River: July 8, Nandua Creek: July 9), and fall of 2019 (834–867 days old; York River: Oct. 21, Choptank River: Oct. 28, Nandua Creek: Oct. 28). Deployment in June of 2018 and each of the four consecutive sampling times will be referred to as T0, T1, T2, T3, and T4, respectively. Assessment of survival involved counting live oysters and removing empty shells. While units were assessed for survival, oysters were sampled for whole live weight without replacement. For the first assessment in the fall of 2018 (T1), survival within each unit was calculated as live oysters divided by the number of oysters deployed. For all subsequent assessments, survival within each unit was calculated up to time *t* as:

$${\text{Survival}}_{t } = \frac{{{\text{Live}}_{t} }}{{{\text{Live}}_{t - 1} - {\text{Samples Removed}}_{t - 1} }} \times {\text{Survival}}_{t - 1} ,$$

where *t* indicates the sampling time and Samples Removed is the number of live oysters sampled without replacement.

Two survival traits were analyzed in this study: (i) survival during the time of an expected mortality event, i.e., between the April/May sampling and the early July sampling (T2–T3), which was defined as late spring survival and calculated as the number of live oysters in early July divided by the number of live oysters in the units in April/May (post-sampling); and ii) final survival, defined as survival between deployment and the final sampling in the fall of 2019 (T0–T4).

### Weight

Families were measured for whole live weight twice during the field test as they were assessed for survival: spring weight in April/May 2019 (T2) and final weight in fall of 2019 (T4). Shells were cleaned of sediment and fouling organisms before weighing. For triploid families, 15 individuals were sampled per unit (bag or basket) for both spring and final weight. For tetraploid families, 15 individuals were sampled per unit for spring weight and 10 individuals were sampled per unit for final weight.

### Genetic analyses

Survival and weight data from triploid and tetraploid families were analyzed using linear mixed models in ASReml-R (VSNi, Hemel Hempstead, UK) [38]. Survival within units was converted to binary data for individual animals (dead = 0, alive = 1) for the linear mixed models. For survival or weight in triploid families, the following linear mixed model was used:

1 $${\mathbf{y}} = \, \upmu \, + {\text{ Line }} + {\text{ Unit}}_{{{\text{Family}}}} + {\text{ Animal }} + \varepsilon,$$

where **y** is a vector of measurements specific to a site (e.g., survival at YR), µ is the mean of the measurements, Line is a fixed effect of paternal and maternal line, Unit_Family_ is a random effect indicating unit nested within family, Animal is the random genetic effect linked to the pedigree, and ε is the residual variation. Data from tetraploid families were analyzed with the following linear mixed model:

2 $${\mathbf{y}} = \, \upmu \, + {\text{ Spawn }} + {\text{ Line }} + {\text{ Unit}}_{{{\text{Family}}}} + {\text{ Family }} + {\text{ Animal }} + \varepsilon,$$

where Spawn is a fixed effect indicating whether the individual was spawned in June or July of 2017, and Family represents the random effect of the full-sib family (non-additive genetic effects), sire/maternal effects, and common environment effects. Models with triploids omitted Family because triploids were reared in units of half-sib families which prevented estimation of genetic effects related to the full-sib family.

The line effect in the mixed models specified the proportional derivation of individuals from each line. For example, triploids made from the (tetraploid) 4GEN line and the (diploid) DBY_LEW line were assigned a value of 0.66 for 4GEN and 0.33 for DBY_LEW (see Additional file 3, Table S3). Line was treated as a set of covariates using the grp(obj) argument in ASReml-R [38]. For models on triploid populations, diploid and tetraploid lines were in the grp(obj) argument, whereas in models for tetraploid populations, only tetraploid lines were included. Spawn was excluded in models with triploids because the maternal diploid line and spawn date were confounded.

All genetic analyses linked the Animal term in the mixed model to a pedigree using an inverse additive relationship matrix. Inverse additive relationship matrices were produced using the polyAinv package in R [33], which applies rules that are appropriate for pedigrees with different ploidies, including odd-numbered ploidies [33]. The input for polyAinv was an 8-column pedigree, with the 8th column accounting for probabilistic contributions of each dam to triploid families [39] (see Additional file 4: Table S4). Triploids were assumed to have equal contributions from 5 unrelated dams, and thus each triploid was assigned a “0.2” probability of deriving from each diploid. The probability of double reduction in tetraploids in which two sister chromatids segregate to the same gamete [40], was set to 0 for the sire gamete (“sire lambda”) and the dam gamete (“dam lambda”) because double reduction was assumed absent in this analysis. However, to examine the effect of double reduction on results, which have been estimated to occur at a frequency of 0.074 in Pacific oysters [41], univariate models for survival and weight were also fit with sire lambda values set at 0.074 for all triploids in the analysis, as well as sire and dam lambda values set at 0.074 for all tetraploids in the analysis. ASReml “ginverse” objects (.giv files) were produced from polyAinv and used in ASReml-R as inverse additive relationship matrices.

Narrow-sense heritabilities (h^2^) were estimated for late spring survival (T2–T3), final survival (T0 to T4), spring weight (T2), and final weight (T4) in each population. Herein, population refers to a combination of ploidy and site (e.g., triploid families at York River). Narrow-sense heritabilities on the observed scale (${\text{h}}_{{\text{o}}}^{{2}} )$ were calculated with the following equation in triploid families:

$${\text{h}}_{{\text{o}}}^{{2}} { = }\frac{{\upsigma_{{\text{a}}}^{{2}} }}{{\upsigma_{{\text{a}}}^{{2}} { + }\upsigma_{{\text{u}}}^{{2}} { + }\upsigma_{{\upvarepsilon }}^{{2}} }},$$

And with the following equation in tetraploid families:

$${\text{h}}_{{\text{o}}}^{{2}} { = }\frac{{\upsigma_{{\text{a}}}^{{2}} }}{{{ }\upsigma_{{\text{a}}}^{{2}} { + }\upsigma_{{\text{u}}}^{{2}} { + }\upsigma_{{\text{f}}}^{{2}} { + }\upsigma_{{\upvarepsilon }}^{{2}} }},$$

where $\upsigma_{{\text{a}}}^{{2}}$, $\upsigma_{{\text{u}}}^{{2}}$, $\upsigma_{{\upvarepsilon }}^{{2}}$, and $\upsigma_{{\text{f}}}^{{2}}$ represent the estimated variance for Animal, Unit, Residual, and Family effects, respectively, from Eqs. (1) and (2). Standard errors were calculated using vpredict in ASReml-R. For survival traits, heritability estimates were adjusted to the underlying scale (${\text{h}}_{{\text{u}}}^{{2}}$) using the following equation from Dempster and Lerner [42]:

$${\text{h}}_{{\text{u}}}^{{2}} = {\text{h}}_{{\text{o}}}^{{2}} \frac{{\left( {p\left( {1 - p} \right)} \right)}}{{z^{2} }}$$

where *p* is the proportion survival and *z* is the height of the standard normal curve corresponding to *p*. The value for *p* was the average survival for the data set being analyzed. The *z* value was calculated with the following commands in R [43]:

$$a = {\text{ qnorm }}(p),$$

$$z = {\text{ dnorm }}(a),$$

where *a* is the *z* score of the normal distribution corresponding to *p*, qnorm is a quantile function of the normal distribution, and dnorm is a density function of the normal distribution. Standard errors were calculated for heritabilities on the underlying scale using the following equation (e.g., [26]):

$$\left( {\frac{{{\text{h}}_{{\text{u}}}^{{2}} }}{{{\text{h}}_{{\text{o}}}^{{2}} }}} \right){\text{h}}_{{{\text{ose}}}}^{2} ,$$

where ${\text{h}}_{{{\text{ose}}}}^{2}$ is the standard error of the heritability on the observed scale.

Bivariate versions of Eqs. (1) and (2) were used to estimate genetic and phenotypic correlations. Correlations were estimated between survival of triploid populations, survival of triploid and tetraploid populations, and between weight and survival within each population. Between triploid families at different sites, a bivariate version of Eq. (1) was used with covariances for Residual and Unit effects set to 0. Correlations between triploid and tetraploid families were estimated with a bivariate version of Eq. (2), with variance for the Family effect in triploid families set to 0 and covariances set to 0 for Residual, Unit, and Family effects. Correlations between weight and survival within populations were estimated with covariances set to 0 for Residual effects. For bivariate models consisting of triploid and tetraploid populations, all levels of Line (diploid and tetraploid) were modeled and Spawn was excluded.

Based on the resulting variance component estimates, genetic correlations were estimated as:

$${\text{r}}_{{{\text{g }}\left( {1,2} \right)}} { = }\frac{{\upsigma_{{\text{a(1, 2)}}} }}{{{ }\sqrt {\upsigma_{{{\text{a1}}}}^{{2}} } { }\sqrt {\upsigma_{{{\text{a2}}}}^{{2}} } }},$$

where $\upsigma_{{\text{a (1, 2)}}}$ represents the covariance in the Animal term for traits 1 and 2, and $\upsigma_{{{\text{a1}}}}^{{2}}$ and $\upsigma_{{{\text{a2}}}}^{{2}}$ represent the estimated variance of the Animal term for traits 1 and 2, respectively. Standard errors were estimated using vpredict in ASReml-R. Phenotypic correlations between survival and weight within tetraploid populations were estimated as:

$${\text{r}}_{{{\text{p }}\left( {1,2} \right)}} { = }\frac{{\upsigma_{{\text{a (1, 2)}}} { + }\upsigma_{{\text{u (1, 2)}}} { + }\upsigma_{{\text{f (1, 2)}}} { }}}{{{ }\sqrt {{ }\upsigma_{{{\text{a1}}}}^{{2}} { + }\upsigma_{{{\text{u1}}}}^{{2}} { + }\upsigma_{{{\text{f1}}}}^{{2}} { + }\upsigma_{{{\varepsilon 1}}}^{{2}} { }} { }\sqrt {{ }\upsigma_{{{\text{a1}}}}^{{2}} { + }\upsigma_{{{\text{u2}}}}^{{2}} { + }\upsigma_{{{\text{f2}}}}^{{2}} { + }\upsigma_{{{\varepsilon 2}}}^{{2}} { }} }},$$

where $\upsigma_{{\text{u (1, 2)}}}$, and $\upsigma_{{\text{f (1, 2)}}}$ represent the estimated covariance between traits 1 and 2 for Unit and Family terms, respectively, $\upsigma_{{{\text{u1}}}}^{{2}}$, $\upsigma_{{{\text{f1}}}}^{{2}}$, and $\upsigma_{{{\varepsilon 1}}}^{{2}}$ represent the estimated variance for trait 1 for Unit, Family, and Residual terms, respectively, while $\upsigma_{{{\text{u2}}}}^{{2}}$, $\upsigma_{{{\text{f2}}}}^{{2}}$, and $\upsigma_{{{\varepsilon 2}}}^{{2}}$ represent the estimated variance for trait 2 for Unit, Family, and Residual terms, respectively. Terms associated with family were omitted for estimating phenotypic correlations in triploid populations.

## Results

### Families produced

Thirty-nine of the 46 tetraploid families and 20 of the 23 triploid families had sufficient numbers of oysters to be deployed in the field trial: 20 tetraploid and 10 triploid families came from the first spawn (June 2017), and 19 tetraploid families and 10 triploid families came from the second spawn (July 2017). For triploid families, the tetraploid sires were mostly from 4GEN (5), 4OBOY (5), and 4LGT (5), and to a lesser extent from 4GNL (3) and 4VBOY (2) (Table 1). All tetraploid sires of deployed triploid families were common to a tetraploid family that was also deployed. Four of the deployed tetraploid families did not share a sire with a triploid family that was deployed.

**Table 1:** Broodstock of triploid and tetraploid families

Line	3N Fams		4N Fams	
	Sire	Dam	Sire	Dam
*Spawn 1*				
DBY_LEW	–	10	–	–
DBY_LYN	–	0	–	–
4GEN	3	–	4	5
4GNL	1	–	2	5
4VBOY	1	–	4	3
4OBOY	3	–	5	4
4LGT	2	–	5	3
*Spawn 2*				
DBY_LEW	–	0	–	–
DBY_LYN	–	10	–	–
4GEN	2	–	3	5
4GNL	2	–	3	0
4VBOY	1	–	2	0
4OBOY	2	–	4	8
4LGT	3	–	7	6

Number of individuals from each line used as broodstock for triploid (3N) and tetraploid (4N) families (Fams) of *Crassostrea virginica* spawned on either June 14, 2017 (Spawn 1) or July 10, 2017 (Spawn 2) for the field trial. DBY_LEW and DBY_LYN are diploid lines; 4GEN, 4GNL, 4VBOY, 4OBOY, and 4LGT are tetraploid lines. All sires used to make triploid families were also used to make tetraploid families. The number of sires indicated for tetraploid families includes those used to make triploid families

### Ploidy

All samples from triploid families were verified triploid. In one tetraploid family, 2 of 15 sampled were triploid, and in another tetraploid family, 1 of 15 was diploid. All other tetraploid families were certified as 100% tetraploid, including the tetraploid families that made up 4OBLT and all samples (25/25) from 4GEN, 4GNL, and 4VBOY.

### Environmental conditions

**Fig. 2:**
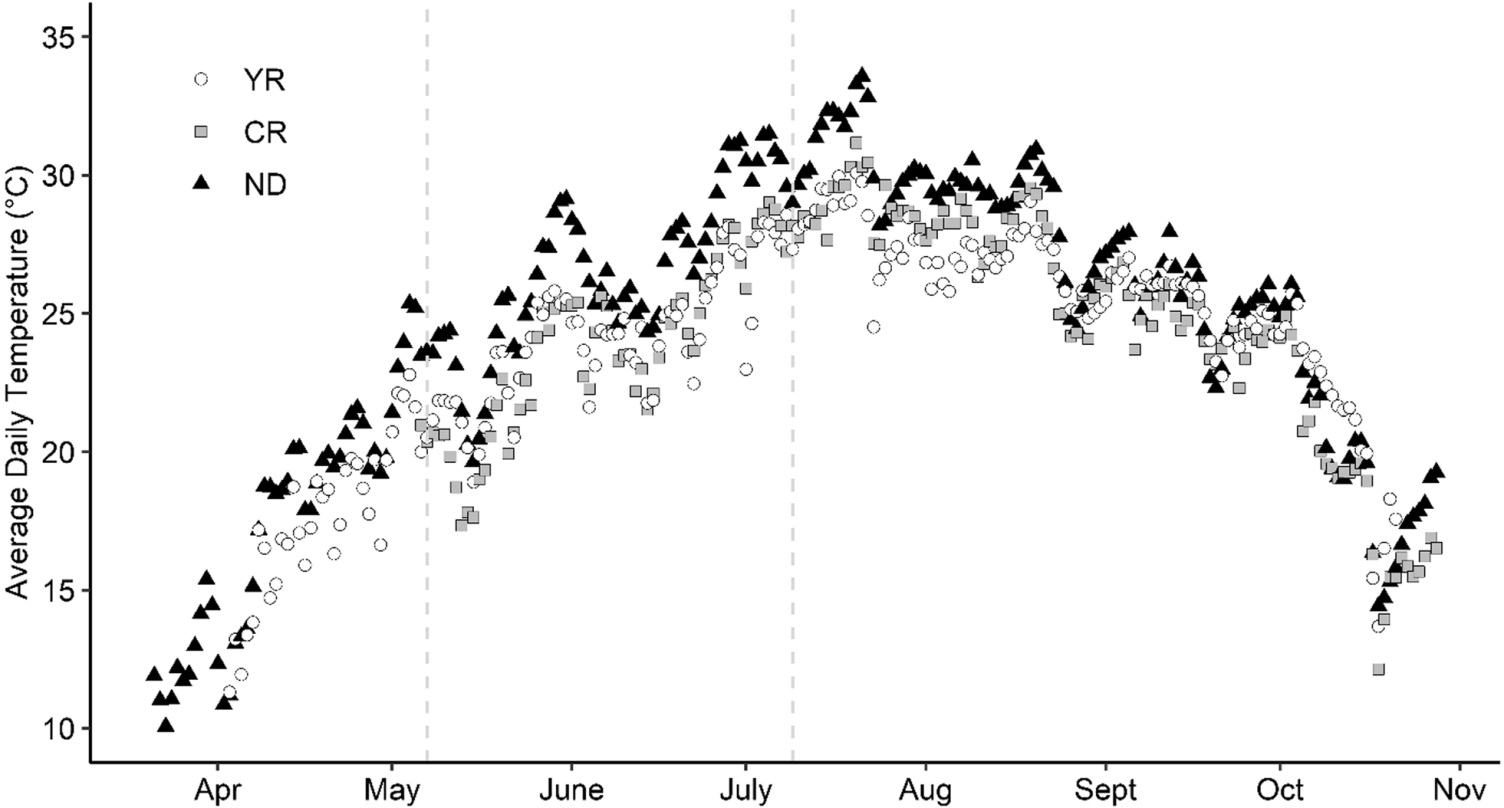
Average daily temperature. Average daily temperature (degrees Celsius) from March 21st, 2019, to October 28th, 2019, at York River (YR), Choptank River (CR), and Nandua Creek (ND). Temperature data were collected at each site by HOBO^®^ temperature loggers. Dotted lines represent the survival assessments in spring and summer of 2019

Temperature loggers collected data from April 3 to October 21 at York River, from May 6 to October 28 at Choptank River, and from March 21 to October 28 at Nandua Creek (Fig. 2). For the time when data were available at all sites (May 6 to October 21), mean average daily temperature was 24.9 °C at York River, 24.7 °C at Choptank River, and 26.5 °C at Nandua Creek. Between the survival assessment in spring 2019 and summer 2019 (May 7 to July 9), mean average daily temperature was 24.1 °C at York River, 23.8 °C at Choptank River, and 26.5 °C at Nandua Creek.

**Fig. 3:**
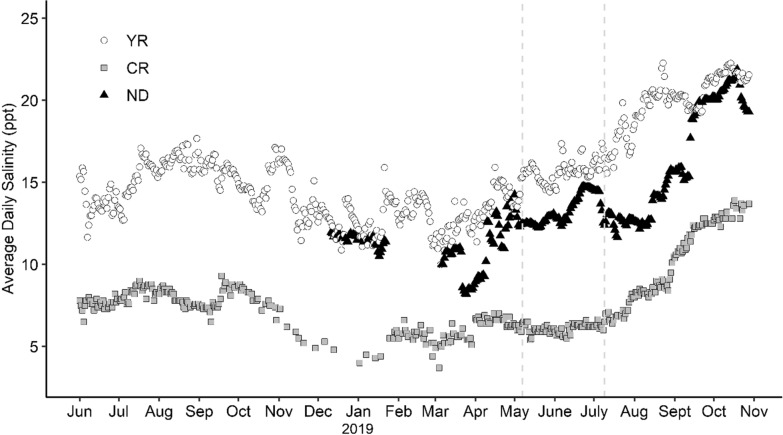
Average daily salinity. Average daily salinity (ppt) from June 1st, 2018, to October 28th, 2019, at York River (YR), Choptank River (CR), and Nandua Creek (ND). Salinity for YR is from Virginia Estuarine and Coastal Observing System monitoring station in Gloucester Point, Virginia (YRK005.40). Salinity for CR is from Horn Point Laboratory, University of Maryland, in Cambridge, Maryland. Salinity for ND collected by HOBO^®^ conductivity logger. Dotted lines represent the survival assessments in spring and summer of 2019

Salinity data were collected at different times at each site. For Nandua Creek, salinity data were collected from December 11, 2018, to October 28, 2019, with no data between January 22, 2019, and March 6, 2019, because of mechanical failure of the logger. Salinity data from York River and Choptank River were available from June 1, 2018, to October 28, 2019. From the time range when data were generally available at all sites (December 11, 2018–October 28, 2019), mean average daily salinity was 15.9 ppt at York River, 7.7 ppt at Choptank River, and 13.7 ppt at Nandua Creek (Fig. 3). Salinity was lowest at each site between November 2018 and April 2019, when the mean average daily salinity was 13.0 ppt at York River, 10.9 ppt at Nandua Creek, and 5.5 ppt at Choptank River.

### Survival

Across all sites, late spring survival (T2–T3) ranged from 0.89 to 1.0 among diploid reference lines and from 0.81 to 0.96 in tetraploid reference lines (Table 2). Late spring survival ranged from 0.60 to 1.0 in triploid families at Nandua Creek, from 0.79 to 1.0 in triploid families at Choptank River, from 0.91 to 1.0 in triploid families at York River, and from 0.64 to 1.0 in tetraploid families at York River. Tetraploid families in the Choptank River had low survival between fall of 2019 (T1) and spring of 2019 (T2), with 100% mortality in many families. The mortality occurred while the oysters were overwintering in “Taylor” floats and the salinity was low (< 7 ppt). As a result of the mortality, the field test for the tetraploid families at Choptank River was terminated in winter of 2019 and, therefore, no data from tetraploid families at Choptank River were analyzed.

**Table 2:** Late spring and final survival

Group	York River			Choptank River			Nandua Creek		
	Min	Mean	Max	Min	Mean	Max	Min	Mean	Max
*Late spring survival (T2–T3)*									
2N Lines	0.94	0.97	1.0	0.93	0.94	0.94	0.89	0.93	0.96
4N Lines	0.81	0.88	0.96	–	–	–	0.87	0.88	0.90
3N Families	0.91	0.97	1.0	0.79	0.94	1.0	0.60	0.88	1.0
4N Families	0.64	0.91	1.0	–	–	–	–	–	–
*Final survival (T0–T4)*									
2N Lines	0.72	0.78	0.82	0.50	0.68	0.79	0.77	0.84	0.9
4N Lines	0.27	0.46	0.73	0.00	0.08	0.24	0.48	0.63	0.75
3N Families	0.60	0.76	0.92	0.32	0.49	0.89	0.50	0.77	0.99
4N Families	0.10	0.53	0.77	–	–	–	–	–	–

Minimum, mean, and maximum late spring survival and final survival in diploid lines, tetraploid lines, triploid families, and tetraploid families of *Crassostrea virginica* measured at three sites in the Chesapeake Bay (York River, Choptank River, Nandua Creek). Lines or families with an average of less than 50 animals per replicate were excluded from calculation of minimum, mean, and maximum. Late spring survival was not calculated in tetraploid lines at CR because all tetraploid lines had less than 50 individuals per replicate by spring of 2019. 2N = diploids, 3N = triploids, 4N = tetraploids, T0 = deployment in spring of 2018, T2 = spring of 2019, T3 = summer of 2019, T4 = fall of 2019

**Fig. 4:**
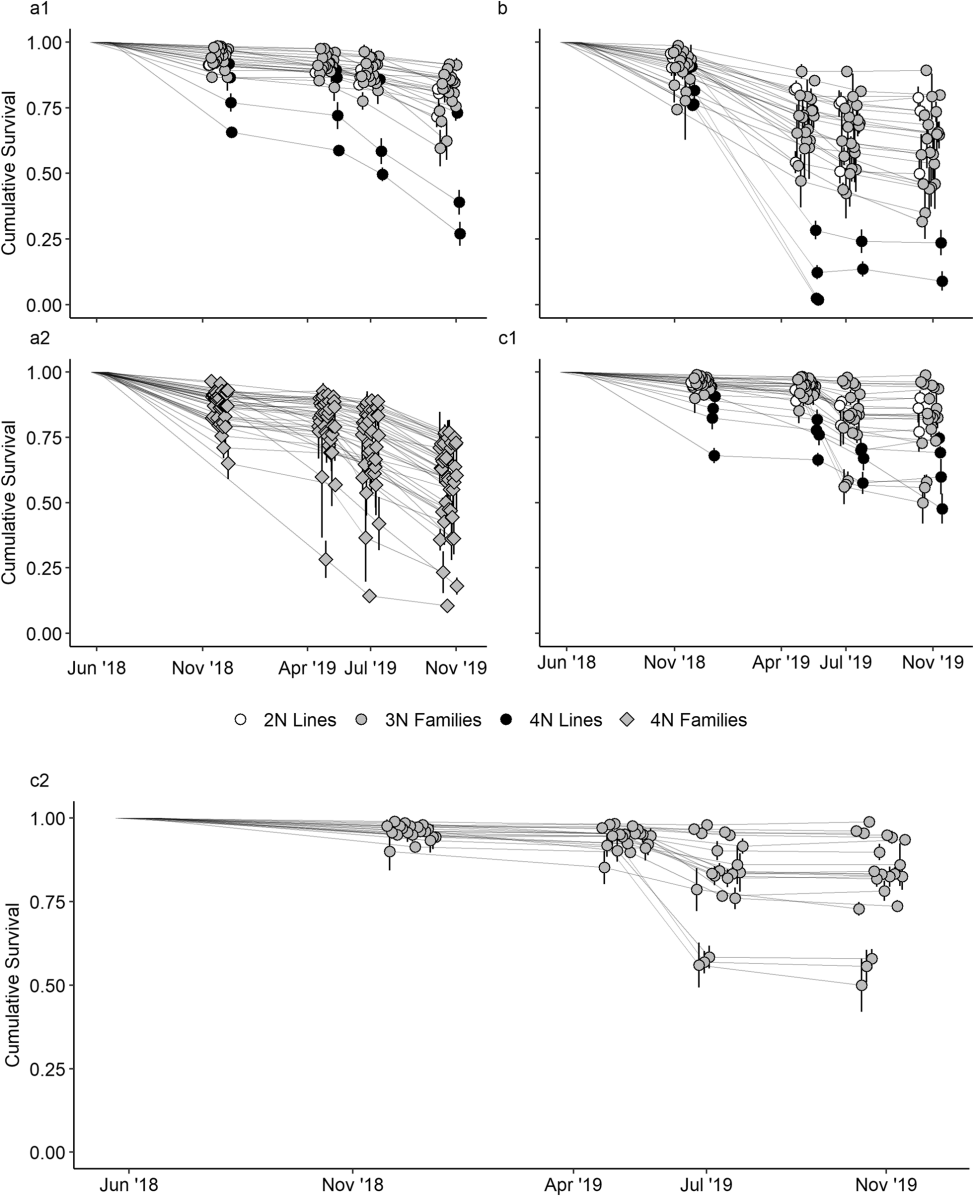
Survival in lines and families. Survival in diploid lines, triploid families, and tetraploid lines from June 2018 to November 2019 at (**a**) York River, (**b**) Choptank River, and (**c**) Nandua Creek. Diploid lines, triploid families, and tetraploid lines are plotted separately (**a1**) from tetraploid families (**a2**) at the York River. Triploid families at Nandua Creek are plotted among diploid lines and tetraploid lines (**c1**) and plotted separately (**c2**). Error bars represent standard error. 2N = diploid, 3N = triploid, 4N = tetraploid, Points jittered horizontally for visualization

Final survival (T0–T4) was higher in diploids and triploids than in tetraploids (Fig. 4; Table 2). For the York River deployment, mean survival was 0.78 in the diploid reference lines, 0.76 in the triploid families, 0.53 in the tetraploid families, and 0.46 in the tetraploid reference lines. At the Choptank River, mean survival was 0.68 in the diploid reference lines, 0.49 in the triploid families, and 0.08 in the tetraploid reference lines. For Nandua Creek, mean survival was 0.84 in diploid reference lines, 0.77 in triploid families, and 0.63 in tetraploid reference lines.

Variance of final survival among diploid reference lines was higher in lines selected for low salinity than among lines selected for high salinity (see Additional file 5: Table S5). At Choptank River (low salinity), mean survival was 0.79 for LILY, 0.74 for LFAMS, and 0.50 for LOLA. At York River (high salinity), mean survival was 0.82 for XB, 0.80 for HNRY, and 0.72 for DBY. At Nandua Creek (also high salinity), mean survival was 0.90 for HNRY, 0.86 for DBY, and 0.77 for XB.

Final survival was more variable in tetraploid reference lines than in diploid reference lines (see Additional file 5: Table S5). At York River, mean survival was 0.73 for 4OBLT, 0.39 for 4GNL, and 0.27 for 4GEN. Survival of 4VBOY at York River was not calculated because of human errors in collecting the data. At Choptank River, 4OBLT had the highest mean survival (0.24), with 0.09 for 4GNL and 0 for 4GEN and 4VBOY. 4OBLT also had the highest mean survival at Nandua Creek (0.75), with 0.60 for 4GNL, 0.48 for 4GEN, and 0.69 for 4VBOY.

There was high variance in survival among triploid families and among tetraploid families at all sites by the end of the field trial (Fig. 4; Table 2). For triploid families, the range in survival was highest at Choptank River (0.32–0.89), followed by Nandua Creek (0.50–0.99) and York River (0.60–0.92). Survival in tetraploid families at York River ranged from 0.10 to 0.77.

Extent and timing of mortality in families differed among sites. In the York River, tetraploid and triploid families had the highest mortality between summer of 2019 (T3) and fall of 2019 (T4), resulting in a decrease of the mean survival by 0.15 and 0.08, respectively. In the Choptank River, the greatest mortality in the triploid families was between fall of 2018 (T1) and spring of 2019 (T2), when mean survival decreased by 0.21. For Nandua Creek, highest mortality in triploid families occurred during the ‘late spring survival period’ between spring of 2019 (T2) and summer of 2019 (T3), when mean survival decreased by 0.11 and four families had survival less than 0.80 (Fig. 4).

### Weight

Across all sites, mean spring weight (spring 2019; T2) ranged from 27.6 to 48.8 grams (g) among triploid and tetraploid families (Table 3). Triploids at Nandua Creek had the highest mean weight (48.8 g ± 12.2 [standard deviation]) followed closely by triploids at York River (47.2 g ± 11.5). The lowest mean spring weight was in triploids at Choptank River (27.6 g ± 8.3). The coefficient of variation of weight was highest in triploids at Choptank River (0.30) and lowest in triploids at York River (0.24).

**Table 3:** Spring and final weight of triploid and tetraploid families

	Spring weight (T2)				Final weight (T4)			
	4N YR	3N YR	3N CR	3N ND	4N YR	3N YR	3N CR	3N ND
Mean	38.4	47.2	27.6	48.8	74.5	118.3	66.5	87.3
SD	10.3	11.5	8.3	12.2	19.9	24.4	18.5	20.9
CV	0.26	0.24	0.30	0.25	0.27	0.21	0.28	0.24

Mean weight, standard deviation (SD), and coefficient of variation (CV) for triploid (3N) and tetraploid (4N) families of *Crassostrea virginica* reared at three sites in the Chesapeake Bay (York River, Choptank River, Nandua Creek) when measured in the spring and fall of 2019. Weight reported in grams. T2 = spring of 2019, T4 = fall of 2019

Across all sites, mean final weight (fall 2019; T4) ranged from 66.5 to 118.3 g among triploid and tetraploid families (Table 3). Mean final weight was highest in triploids at York River (118.3 g ± 24.4) and lowest in triploids at Choptank River (66.5 g ± 18.5). The coefficient of variation of weight was highest in triploids at Choptank River (0.28) and lowest in triploids at York River (0.21). Mean final weight in triploids at York River was much higher than in tetraploids at the York River (118.3 g vs. 74.5 g).

### Genetic analysis of survival

For late spring survival (T2–T3), estimates of narrow-sense heritability on the underlying scale ranged from 0.04 to 0.57 across populations (Table 4). The estimate of narrow-sense heritability on the underlying scale was 0.15 ± 0.10 (standard error) for tetraploid families at York River, 0.04 ± 0.08 for triploid families at York River, 0.13 ± 0.12 for triploid families at Choptank River, and 0.57 ± 0.23 for triploid families at Nandua Creek.

**Table 4:** Estimates of variance components and heritabilities of survival

	4N_YR	3N_YR	3N_CR	3N_ND
*Late spring survival (T2–T3)*				
${\upsigma }_{{\text{a}}}^{{2}}$	0.005	< 0.001	0.003	0.015
${\upsigma }_{{\text{u}}}^{{2}}$	0.010	0.001	0.003	0.001
${\upsigma }_{{\text{f}}}^{{2}}$	< 0.001	–	–	–
${\upsigma }_{{\upvarepsilon }}^{{2}}$	0.076	0.033	0.059	0.083
obs h^2^	0.05 (0.04)	0.01 (0.02)	0.05 (0.05)	0.15 (0.06)
und h^2^	0.15 (0.10)	0.04 (0.08)	0.13 (0.12)	0.57 (0.23)
*Final survival (T0–T4)*				
${\upsigma }_{{\text{a}}}^{{2}}$	0.018	0.001	0.026	0.034
${\upsigma }_{{\text{u}}}^{{2}}$	0.012	0.006	0.015	0.003
${\upsigma }_{{\text{f}}}^{{2}}$	0.003	–	–	–
${\upsigma }_{{\upvarepsilon }}^{{2}}$	0.206	0.170	0.196	0.129
obs h^2^	0.08 (0.05)	0.004 (0.02)	0.11 (0.07)	0.21 (0.08)
und h^2^	0.13 (0.08)	0.006 (0.04)	0.18 (0.12)	0.42 (0.17)

Estimates of additive genetic variation (${\upsigma }_{{\text{a}}}^{{2}}$), variation from unit effect (${\upsigma }_{{\text{u}}}^{{2}}$), variation from family effect (${\upsigma }_{{\text{f}}}^{{2}}$), residual variation (${\upsigma }_{{\upvarepsilon }}^{{2}}$), and narrow-sense heritability (h^2^) on the observed (obs) and underlying scale (und) for late spring survival and final survival for triploid (3N) and tetraploid (4N) families of *Crassostrea virginica* measured at three sites in the Chesapeake Bay (York River, Choptank River, Nandua Creek). Standard errors are in parentheses. “–” indicates variable not in model. T0 = deployment in spring of 2018, T2 = spring of 2019, T3 = summer of 2019, T4 = fall of 2019

The range in line effect estimates for late spring survival was highest in triploids at Nandua Creek (see Additional file 6: Table S6A). For triploids, line effect estimates ranged from − 0.03 to 0.05 at the York River and from − 0.05 to 0.16 at Choptank River. Line effect estimates for tetraploids at the York River ranged from − 0.10 to 0.13. In triploids at Nandua Creek, 4GNL had the most negative estimated effect of − 0.33 ± 0.08, closely followed by 4VBOY, which had an effect estimate of − 0.29 ± 0.09. 4LGT had the most positive effect estimate for triploids at Nandua Creek with 0.06 ± 0.07.

Estimates of genetic correlations between populations for late spring survival ranged from − 0.57 to 0.18 (Table 5). The genetic correlation between triploid families at Choptank River and Nandua Creek was estimated at − 0.31 ± 0.33 and that between tetraploid families at York River and triploid families at Nandua Creek at 0.18 ± 0.53. The genetic correlation between tetraploid families at York River and triploid families at Choptank River was estimated at − 0.57 ± 0.66. No genetic correlations were estimated between triploids at York River and other populations because the additive genetic variation was markedly low for late spring survival of triploids at York River.

**Table 5:** Estimates of genetic correlations for survival

		Estimate
*Late spring survival (T2–T3)*		
3N Nandua Creek	3N Choptank River	− 0.31 (0.33)
3N Nandua Creek	4N York River	0.18 (0.53)
3N Choptank River	4N York River	− 0.57 (0.66)
*Final survival (T0–T4)*		
3N Nandua Creek	3N Choptank River	− 0.50 (0.23)
3N Nandua Creek	4N York River	0.05 (0.39)
3N Choptank River	4N York River	0.15 (0.47)

Estimates of genetic correlations for late spring survival and final survival between triploid (3N) families and triploid and tetraploid (4N) families measured at three sites in the Chesapeake Bay (York River, Choptank River, and Nandua Creek). Standard errors are in parentheses. Note: bivariate models not fit for 3N York River due to low additive genetic variance for late spring and final survival. T0 = deployment in spring of 2018, T2 = spring of 2019, T3 = summer of 2019, T4 = fall of 2019

Estimates of narrow-sense heritability on the underlying scale ranged from 0.006 to 0.42 for final survival (T0–T4) across populations (Table 4). The estimate of narrow-sense heritability on the underlying scale was 0.13 ± 0.08 for tetraploid families at York River, 0.006 ± 0.04 for triploid families at York River, 0.18 ± 0.12 for triploid families at Choptank River, and 0.42 ± 0.17 for triploid families at Nandua Creek.

The range in line effect estimates for final survival was highest for triploids at Choptank River (see Additional file 6: Table S6B). Line effect estimates ranged from − 0.26 to 0.33 for triploids in York River, from − 0.21 to 0.46 for triploids in Choptank River, from − 0.32 to 0.17 for triploids at Nandua Creek, and from − 0.17 to 0.19 for tetraploids in York River.

Estimates of genetic correlations for final survival ranged from − 0.50 to 0.15 (Table 5). Between triploid families at Choptank River and Nandua Creek, the genetic correlation estimate was − 0.50 ± 0.23. The estimate of the genetic correlation between triploid families at Nandua Creek and tetraploid families at York River was 0.05 ± 0.39, and that between triploid families at Choptank River and tetraploids at York River was 0.15 ± 0.47. No genetic correlations were estimated between triploids at York River and other populations because the additive genetic variation was notably low for final survival in triploids at York River.

Univariate models for late spring survival and final survival when assuming a frequency of double reduction of 0.074 returned similar results as those reported above, which assumed no double reduction (see Additional file 7: Table S7A).

### Genetic analysis of weight

Estimates of narrow-sense heritability for spring weight (T2) ranged from < 0.001 to 0.60. The estimate of narrow-sense heritability was 0.28 ± 0.14 for tetraploid families at York River, 0.60 ± 0.20 for triploid families at York River, 0.07 ± 0.09 for triploid families at Choptank River, and < 0.001 ± 0.08 for triploid families at Nandua Creek (Table 6).

**Table 6:** Estimates of variance components and heritabilities of weight

	4N_YR	3N_YR	3N_CR	3N_ND
*Spring weight (T2)*				
${\upsigma }_{{\text{a}}}^{{2}}$	27.76	72.25	4.09	< 0.001
${\upsigma }_{{\text{u}}}^{{2}}$	14.06	5.04	4.92	18.16
${\upsigma }_{{\text{f}}}^{{2}}$	0.71	–	–	–
${\upsigma }_{{\upvarepsilon }}^{{2}}$	58.28	43.39	50.34	118.62
h^2^	0.28 (0.14)	0.60 (0.20)	0.07 (0.09)	< 0.001 (0.08)
*Final weight (T4)*				
${\upsigma }_{{\text{a}}}^{{2}}$	32.05	269.27	116.04	44.75
${\upsigma }_{{\text{u}}}^{{2}}$	34.06	16.80	35.43	17.90
${\upsigma }_{{\text{f}}}^{{2}}$	40.79	–	–	–
${\upsigma }_{{\upvarepsilon }}^{{2}}$	237.60	277.02	144.02	312.30
h^2^	0.09 (0.15)	0.48 (0.18)	0.39 (0.19)	0.12 (0.09)

Estimates of additive genetic variation (${\upsigma }_{{\text{a}}}^{{2}}$), variation from unit effect (${\upsigma }_{{\text{u}}}^{{2}}$), variation from family effect (${\upsigma }_{{\text{f}}}^{{2}}$), residual variation (${\upsigma }_{{\upvarepsilon }}^{{2}}$), and narrow-sense heritability (h^2^) for spring weight and final weight for triploid (3N) and tetraploid (4N) families of *Crassostrea virginica* measured at three sites in the Chesapeake Bay (York River, Choptank River, Nandua Creek). Standard errors are in parentheses. “–” indicates variable not in model. T2 = spring of 2019, T4 = fall of 2019

Estimates of narrow-sense heritability for final weight (T4) ranged from 0.09 to 0.48. The estimate was 0.09 ± 0.15 for tetraploid families at York River, 0.48 ± 0.18 for triploid families at York River, 0.39 ± 0.19 for triploid families at Choptank River, and 0.12 ± 0.09 for triploid families at Nandua Creek (Table 6).

Univariate models for spring weight and final weight assuming a frequency of double reduction of 0.074 gave similar results to assuming no double reduction (see Additional file 7: Table S7B). Estimated line effects from univariate models on spring weight and final weight are in supplementary material (see Additional file 6: Table S6A, B).

### Correlations between survival and weight

Correlations between spring weight (T2) and late spring survival (T2–T3) were not estimated due to traits having low additive genetic variation or because of poor model fit. Estimates of heritability on the observed scale were markedly low for late spring survival in triploids at York River (0.01) and spring weight in triploids at Nandua (< 0.001), so bivariate models were not fit for these traits. Results are omitted for genetic correlations between the traits in triploids at Choptank River and tetraploids at York River because of poor model fit.

Correlations were estimated between final weight (T4) and final survival (T0–T4) within triploids at Choptank River, triploids at Nandua Creek, and tetraploids at York River. For triploids at Choptank River, the estimate of the genetic correlation was − 0.40 ± 0.32 and that of the phenotypic correlation was − 0.13 ± 0.11 (Table 7). For triploids at Nandua Creek, the estimate of the genetic correlation was − 0.46 ± 0.30 and that of the phenotypic correlation was − 0.12 ± 0.09. Tetraploids at York River had a genetic correlation estimate of − 0.62 ± 0.26 and a phenotypic correlation estimate of − 0.11 ± 0.07. No correlations were estimated for triploids at York River because the estimate of heritability on the observed scale for final survival was too low (h^2^ = 0.004).

**Table 7:** Estimates of genetic and phenotypic correlations between final survival and weight

	Genetic	Phenotypic
3N Choptank River	− 0.40 (0.32)	− 0.13 (0.11)
3N Nandua Creek	− 0.46 (0.30)	− 0.12 (0.09)
4N York River	− 0.62 (0.26)	− 0.11 (0.07)

Estimates of genetic correlations and phenotypic correlations for final survival (T0 to T4) vs. final weight (T4) within triploid (3N) and tetraploid (4N) families of *Crassostrea virginica* at three sites in the Chesapeake Bay (York River, Choptank River, Nandua Creek. Standard errors are in parentheses. T0 = deployment in spring of 2018, T4 = fall of 2019

## Discussion

### Quantitative genetic analysis of late spring survival at Nandua creek

The primary objective in this study was to examine the genetic basis of late spring mortality events in triploid oysters. These mortality events have typically occurred in May and June and have resulted in the loss of many (> 0.20) near market sized triploid oysters [18, 21, 31]. Over 20 oyster farms in Virginia have reported mortality events matching these criteria since 2012 (Karen Hudson, personal communication), including a farm in Nandua Creek, where the mortality events have occurred several times [21, 31]. Accordingly, survival during the expected time of a mortality event, between May and June, was referred to as late spring survival and treated as a distinct trait for quantitative genetic analysis in this study.

A late spring mortality event occurred within triploid families at Nandua Creek. In late spring at Nandua Creek, defined in this study as between May 7 and June 9, one triploid family had mean survival of 0.79 and three had mean survival less than 0.70. Meanwhile, most of the 20 triploid families had mean survival greater than 0.90. The narrow-sense heritability estimate for late spring survival in triploid families at Nandua Creek was 0.57 ± 0.23 (standard error), implying that variation in survival had a substantial additive genetic basis.

Effects of line of the broodstock were included in the model used for analysis of survival to avoid an upward bias of additive genetic variance. A default assumption in the mixed linear animal model that was used here is that all founder individuals in the pedigree derive from the same randomly bred population. The genetic variance can be overestimated if population structure in the base population is ignored or if relationships among founder individuals are omitted (e.g., [44]). Although the tetraploid and diploid broodstock lines in this experiment derive from the same ancestral line (4GEN or DBY), line was included as an effect in models because each line has a different pedigree. The lines have been mass selected, thus the pedigree and genetic relationships among the parents were unknown. Parents were therefore entered in the pedigree as unrelated. It is possible that relationships or inbreeding among parents biased the estimates of additive genetic variance upward. Such bias could be reduced in future studies by genotyping broodstock individuals to determine genetic relationships among them.

The estimated line effects provide a lead for closer examination of the genetic basis of the late spring mortality event. Line effects from this experiment should be interpreted with caution because the effect of the maternal diploid line and spawn date were confounded in models for triploids and few tetraploid individuals per line were used to make triploid families. Although robust estimates of the effects of individual tetraploid lines could not be produced due to low sample sizes, the effect of the tetraploid line on the late spring mortality event at Nandua Creek was substantial and triploids derived from 4GNL and 4VBOY oysters had much lower late spring survival at Nandua Creek than triploids from other lines. These line effects suggest genetic differences of consequence for late spring survival could exist among the tetraploid lines, and offer a lead for investigation into the genetics of certain tetraploid lines that may increase susceptibility.

Effect of line and high variation in survival of triploid families reared at Nandua Creek were also observed by Ritter [31]. Ritter [31] tested half-sib triploid families that each had a different tetraploid sire and used tetraploids from four of the same lines as used in the current study: 4GEN, 4VBOY, 4GNL, and 4LGT. At the end of the experiment in Ritter [31], cumulative survival among triploid families ranged from 0.22 to 0.88 at Nandua Creek, with most of the mortality occurring in the late spring—between March and June of 2018. Interestingly, high mortality in late spring only occurred in triploid families that derived from 4GEN, 4VBOY, and 4GNL, which corresponds with the origin of the four families at Nandua Creek that had less than 0.80 survival during the late spring in this study. Although Ritter [31] did not estimate genetic parameters, the high variation in survival observed among triploid families in that study also suggests a substantial genetic basis for the trait.

Findings of a genetic basis for late spring survival at Nandua Creek in this study are similar to those for “summer mortality” in diploid *C. gigas* [45, 46]. Dégremont [45] measured summer survival in juveniles (4–6 months old) from 43 full-sib families across three sites along the French coast. Over only a few months, survival ranged from 0.02 to 0.82, resulting in narrow-sense heritability estimates on the observed scale of 0.47 ± 0.20, 0.89 ± 0.40, and 1.08 ± 0.46. In a follow-up study, Dégremont [46] estimated realized heritability of summer survival through divergent selection. “High” (high survival) and “low” (low survival) lines were produced from full-sib oysters that were retained in non-stressful conditions, i.e., separate from the field trials described in Dégremont [45]. Large differences in survival between juveniles in the “high” and “low” lines over two generations resulted in realized heritability estimates ranging from 0.55 ± 0.18 to 1.02 ± 0.20, supporting earlier findings of Dégremont [45].

The findings by Dégremont [45, 46] are pertinent because summer mortality events of *C. gigas* before the widespread impact of Ostreid herpesvirus 1 (OsHV-1), which has been associated with Pacific Oyster Mortality Syndrome (POMS) since 2008 [47–49], parallel late spring mortalities in *C. virginica* by lacking a causative pathogen (*C. virginica*: [18, 21]; *C. gigas*: [50–54]. Additionally, both summer mortality in *C. gigas* and late spring mortality in *C. virginica* occur during the reproductive season and have a hypothesized etiology of a physiological—environmental interaction that involves reproductive effort [21, 53, 55].

The divergent selection lines of Dégremont [45, 46] were the subject of several follow-up studies in attempts to understand the mechanism behind the genetic differences in susceptibility [56–58]. Gonad development was a major factor that distinguished the lines—oysters in the “low” lines often showed a higher percent of gonad area in histological sections and spawning was often “partial” rather than “massive.” However, a mechanism that explains the difference in survival between the divergent lines was not resolved [54]. Investigations of the physiological mechanisms underlying variation in late spring survival in triploid *C. virginica* could benefit from an approach similar to that of Dégremont et al. [46], Huvet et al. [56, 57], and Samain et al. [58]—triploids made from diploid and tetraploid families with the highest and lowest estimated breeding values for late spring survival could be produced and tested to better understand the physiological causes behind the mortalities.

Estimates of genetic correlations provide insight into genetic relationships between late spring survival at Nandua Creek and other traits measured in this experiment. The estimate of the genetic correlation between late spring survival in triploids at Nandua Creek and Choptank River was − 0.31 ± 0.33, indicating there was a genotype by environment interaction (G × E) for the trait between triploids at these two sites. Salinity was likely a major agent of this G × E. Salinity averaged around 6 ppt during the late spring in the Choptank River, which is near the minimum of the range at which *C. virginica* typically exists in the wild (5–40 ppt) [59] and is an uncommonly low salinity for oysters bred by ABC. During this same time, salinity in Nandua Creek averaged around 13 ppt. The low genetic correlation estimates suggest genes associated with susceptibility to a late spring mortality event may largely differ from those influencing the hypothesized low salinity induced mortality at the Choptank River. Similarly, estimates of genetic correlations between survival at Nandua Creek and survival in tetraploids were far from unity. Genetic correlation estimates between triploids at Nandua Creek and tetraploids at York River were low for both late spring survival (0.18 ± 0.53) and final survival (0.05 ± 0.39), suggesting that selecting based on survival of tetraploids will not result in much improved survival of triploids.

A relationship between size and late spring survival was examined in this experiment to address hypotheses that growth rate is a factor in susceptibility to the mortality events. Anecdotal evidence from early reports of the mortality events in the lower Chesapeake Bay was that the fast growing triploids were undergoing mortality [18]. Previous studies have investigated a link between growth and susceptibility by measuring shells of live and dead oysters [18, 21] and found no evidence for such a relationship. The genetic correlation between weight of triploid families at Nandua Creek before the mortality event (spring weight) and late spring survival could not be estimated in this experiment because the heritability for spring weight in triploids at Nandua was very low (h^2^ < 0.001). That late spring survival rather than size was substantially affected by additive genetic effects at Nandua may point to a weak genetic association between the traits. Future studies could yield better insight into the relationship between size and late spring survival by tagging oysters, measuring their size in early spring, and assessing individual survival in the summer.

Although a genetic basis for late spring survival of triploids was observed at Nandua Creek, it is unclear if the selection pressures at Nandua Creek are representative for the more widely observed syndrome of late spring mortalities in triploid *C. virginica*. Several farms in Virginia have reported late spring mortalities in triploids and, although most reports have come from near Nandua Creek on the bayside of the Eastern Shore of Virginia (K. Hudson, personal communication), with presumably similar environments as in Nandua Creek, similar reports have also come from the western section of the Chesapeake Bay and the seaside of the Eastern Shore of Virginia (Atlantic Ocean). Reports of mortality events in triploids have also come from the Gulf Coast of the US (e.g., [60, 61]) and may be similar to the late spring mortalities in the Chesapeake Bay. Estimates of genetic correlations between a late spring mortality event at Nandua and at other site(s) are critical to this understanding.

### Survival of diploids

Several empirical studies, including the present, have evaluated whether diploids are also susceptible to mortality episodes in late spring in the lower Chesapeake Bay. Matt et al. [21] found that diploids of mid-Atlantic genetic origin had low mortality during a late spring mortality event in triploids at Nandua Creek. In the present study, the three diploid reference lines tested had mortality rates of 0.04, 0.06, and 0.11 in late spring, much lower than the mortality observed in the most affected triploid families (0.21, 0.35, 0.40, 0.40). In contrast, Guévélou et al. [18] found high mortality in diploids of mid-Atlantic genetic origin during a late spring mortality event at Nandua Creek, as did Ritter [31] at the nearby Nassawadox Creek. Better insight into survival and its interaction with ploidy may be gained by regularly measuring late spring survival in breeding programs of *C. virginica*.

Survival among diploids involved a comparison between oysters that were derived from mass selection and family breeding. Three of the diploid reference lines tested in this study were ABC mass-selected lines [24] (LOLA, DBY, and XB) and three were products of family breeding at ABC [26] (LILY, LFAMS, and HNRY). In low salinity, oysters from the family breeding program had substantially higher survival compared to those from the mass-selected lines. In contrast, little difference was found among the diploid lines at the high salinity sites (Nandua Creek and York River). Higher survival was expected in the oysters derived from family breeding because the family breeding program is a continuation of selection of the mass-selected lines. ABC switched to family breeding in diploids in 2015, starting with a base population of families that was founded in large part by the mass-selected lines [26]. At the same time, ABC ceased intensively selecting the mass-selected lines, instead focusing on maintaining their current state through a modified selection regime. Additional trials to compare performance of lines derived from the family breeding program (LILY, HNRY) and mass-selected lines (LOLA, DBY, XB) would be valuable in assessing the effects of family breeding on commercial traits.

### Genetic parameters in triploids and tetraploids

Estimates of heritability in triploid families varied substantially by site. Heritability estimates ranged from 0.006 ± 0.04 to 0.42 ± 0.17 across sites for final survival and from 0.12 ± 0.08 to 0.48 ± 0.18 across sites for final weight. The high range in heritability estimates may be indicative of genotype by site interactions, however it is also probably indicative of low precision of estimates as a result of the relatively small datasets and shallow pedigrees used in this study. Families in this study were part of a two-generation pedigree and created with many tetraploid lines. Future quantitative genetic analyses in polyploids at ABC will benefit from a deeper or genotypic pedigree that can incorporate the effects of lines as genetic groups [62] and produce more robust estimates of genetic parameters.

A consistent pattern within triploid and tetraploid populations was a negative (adverse) estimate of the genetic correlation between final survival and final weight. This indicates that selection for survival in these polyploid populations may involve a sacrifice in growth rate. These results differ from the positive genetic correlations found between total weight and survival in diploid *C. virginica* families at York River and Choptank River [26]. A major caveat in comparing these studies is that data for diploids in Allen et al. [26] were based on several field-tested year classes and hundreds of families, whilst the genetic parameters for triploids and tetraploids in this study represent one year class of only 20 to 39 families. Data from additional year classes of triploids and tetraploids are needed to verify this difference between the genetic correlations in diploid and polyploid populations.

### Data analysis methods

Genetic parameters were estimated for polyploid populations in this study, which required different methods than standard for diploid populations. The analyses in this study relied on adopting methods developed by Gallais [40], Kerr [63], and Hamilton and Kerr [33], which incorporate ploidy in the calculation of coefficients of coancestry, coefficients of inbreeding, and coefficients of relationship. Typically, these coefficients are calculated for and among individuals in a pedigree with methods that assume that each individual is diploid [32]. Incorporating ploidy into the calculations leads to more accurate estimates of the genetic relationships when pedigrees contain polyploids because coancestry and the dynamics of gene flow differ based on ploidy [33, 40, 63]. Calculating genetic relationships based on ploidy is particularly important for pedigrees that contain multiple ploidy levels, as in the current study, because tetraploids and diploids can have unequal genetic contributions to offspring and thus disproportionate genetic relationships with progeny. For example, a tetraploid sire and triploid offspring share a higher proportion of genes than a tetraploid sire and a tetraploid offspring, simply because in the former, 2/3 of the genome of the triploid is contributed by the tetraploid, and in the latter case only 1/2. Thus, if standard methods for diploids were used in this study, the estimated genetic relationship among triploid half-sibs would have been 0.25. Based on the rules proposed by Hamilton and Kerr [33], the relationship was 0.33. Such a difference in estimated genetic relationships influences estimates of genetic parameters and breeding values in a mixed-ploidy pedigree.

The crossing design in this study prioritized estimation of the additive genetic variance derived from tetraploid sires. Creating triploid families by mating each tetraploid to one of two pools of eggs (5 unique dams per pool) enabled additive genetic effects of sires to be estimated over five dams, which has the potential to produce a more robust estimated breeding value for the tetraploid sires than if families were mated using 1 × 1, 1 × 2, or 2 × 2 crossing designs. A consideration, however, is that genetic relationships within and among triploid families were never measured empirically. Triploid families were assumed to contain an equal proportion of individuals descended from each of five diploid dams but the actual proportion of triploids from each dam was unknown. Deviations from the assumed equal contribution from each diploid dam in a triploid family may increase the impact diploid dams had on the additive genetic variation and confound estimates of genetic parameters, although the use of five dams in each pool was expected to provide a buffer to such variation. Nonetheless, the genetic effects of diploid dams can be better accounted for in future studies by rearing full-sib triploid families separately or by reconstructing the pedigree of mass-spawned triploids through genotyping.

### Genetic improvement of triploids

Improving survival and weight in triploids via selective breeding is possible based on the additive genetic variation that was estimated for the traits in this study. However, the process of improving triploids is complicated by the inability to select them directly. Triploid oysters have highly reduced fecundity (e.g., [11, 19, 20], but more problematic is that triploid x triploid crosses result in low survival and almost exclusively aneuploid progeny [64]. Thus, triploids cannot be bred directly, and therefore improvement must come from selection in diploids, tetraploids, or both.

To improve triploid oysters through family breeding, a fundamental question is from which ploidy(s) the phenotypes used for selection should be derived. Table 8 lists three possible breeding approaches and each has trade-offs. The first approach, producing and testing only diploid and tetraploid families for triploid improvement (column a, Table 8) is the most simple approach but also the most uncertain. This approach does not require a pre-existing program for diploid improvement to be altered, and a parallel tetraploid program can proceed independently. The approach is, however, uncertain because both the sign (+/) and size of the response to selection in the triploid traits depends on their genetic correlations with traits in diploids and tetraploids (*rg*_*xy*_ in Table 8, column a), which have yet to be established for commercial traits in any polyploid oyster breeding program. In the second approach (column b, Table 8), diploid and tetraploid families are selected based on the performance of half-sib triploid families, akin to an RRS scheme [28] in which selection is based on performance of half-sib crossbred individuals [27]. This strategy eliminates the dependency on the genetic correlation of traits in triploids with those in diploids and tetraploids. However, selection candidates would be half-sibs of measured individuals, resulting in a lower maximum accuracy of selection than schemes that select full-sibs of measured individuals (*r*_*ux*_ or* r*_*uy*_ in Table 8). The third approach, phenotyping related diploid, triploid, and tetraploid families, is comparable to an RRS scheme in which both purebred and crossbred phenotypes are used for selection (e.g., [29, 30]). Selecting based on phenotypes from all ploidies is the most assured method because it removes the dependency on genetic correlations and allows data on genetically correlated traits to improve the accuracy of selection for triploid performance, but it is also the most laborious approach and it is logistically challenging. If the third approach must be pared down to be practical, only phenotyping the triploids and tetraploids may be the next best option. Assuming mostly additive control of traits, phenotyping tetraploid families may be more important than phenotyping diploid families because the tetraploid parent has a greater genetic contribution in triploid production.

**Table 8:** Possible breeding strategies for improving triploid oysters

	a	b	c	d
Method of selection	Pedigree	Pedigree	Pedigree	Genomic
Produce	2N + 4N	2N + 3N + 4N	2N + 3N + 4N	2N + 3N + 4N
Test	2N + 4N	3N	2N + 3N + 4N	3N
Target trait	3N _*(y)*_	3N _*(y)*_	3N _*(y)*_	3N _*(y)*_
Selected trait	2N or 4N _*(x)*_	3N _*(y)*_	3N _*(y)*_	3N _*(y)*_
Simplified resp. equation	*i* ⋅ *r*_*ux*_ ⋅ *rg*_*xy*_ ⋅* σ*_*ay*_	*i* ⋅* r*_*uy*_ ⋅ *σ*_*ay*_	*i* ⋅* r*_*uy*_ ⋅ *σ*_*ay*_	*i* ⋅* r*_*uy*_ ⋅ *σ*_*ay*_
Max. accuracy (*r*_*ux*_ or* r*_*uy*_)	0.71	0.50	0.71	1

Each strategy (a,b,c, or d) is described by the method of estimating genetic relationships (pedigree-based or genomic-based), the ploidies produced, the ploidies tested (e.g., with a field trial), the trait targeted for improvement, the trait used for breeding value estimates, a simplified version of a response equation, and the theorized maximum possible accuracy (correlation of estimated breeding value and true breeding value) from the breeding scheme. In the example, only two traits exist, a trait in triploids (y) and a trait in diploids or tetraploids (x). *i:* intensity of selection, *r*_*ux*_: accuracy of selection on trait x based on index u for trait x, *r*_*uy*_: accuracy of selection on trait y based on index u for trait y, *rg*_*xy*_: genetic correlation between trait x and trait y, *σ*_*ay*_: additive genetic standard deviation for trait y. Index u refers to estimating breeding values derived from univariate or multivariate animal models

Alternative approaches to pedigree-based family breeding have been proposed and include breeding to maximize heterosis, such as through crosses of inbred lines (e.g., [65]) or interspecific crosses (e.g., [66]). To validate such approaches, future quantitative genetic analyses of triploid oysters should include crossing designs that allow estimation of non-additive genetic variation and of heterosis effects, as has been done in other aquaculture breeding programs (e.g., [30]).

Pedigree-based family breeding can achieve substantial genetic improvement in triploids, but a greater rate of improvement is possible with genomic selection. Genomic selection [67] has been well integrated into breeding of livestock species [68] and has been increasingly applied to aquaculture species [69–71], including oysters (*Crassostrea gigas*: [72, 73]. A major benefit of genomic selection is enabling within-family selection on traits that cannot be directly measured on selection candidates, thereby facilitating selection on these traits with higher accuracy. Importantly, triploid oysters are never selection candidates due to their sterility. Thus, barring very high genetic correlations between traits in triploids and traits in their diploid and tetraploid half-sibs, effectively applying within-family selection for genetic improvement of triploid oysters will only be possible with genomic selection.

Genomic selection has yet to be evaluated in polyploid animals but has been applied in polyploid crops (e.g., [74–77]. Application of genomic selection in polyploid crops has involved addressing complexities of polyploid genomes that make genotyping and estimating allele dosage more challenging (e.g., [75–78]. Applying the methods for genotyping and assessment of inheritance patterns that have been developed for autopolyploid crops (review by [79]) would likely be a good starting place to evaluating genomic selection in polyploid oysters.

## Conclusions

A quantitative genetic analysis among related triploid and tetraploid oysters revealed a substantial genetic basis for a late spring mortality event that affects triploids. The abundant additive genetic variation for the trait suggests selective breeding can reduce the severity of future mortality events in triploids. The estimated effects of tetraploid broodstock lines were not robust but suggest genetic differences in susceptibility to late spring mortality exist among the tetraploid lines tested. Estimates of genetic correlations indicated genotype by environment interactions for triploid survival and weak genetic relationships between survival of tetraploids and triploids at different sites. Phenotyping related triploid and tetraploid families and applying quantitative genetic methods is recommended for genetic improvement of triploid oysters. The application of quantitative genetics for the genetic improvement of triploid oysters in this study offers insight for future research into the breeding of polyploid animals.

## Supplementary Information

Additional file 1: Table S1. Reference lines included in field trial. Table specifying reference lines in field trial.

Additional file 2: Table S2. Deployment of families and reference lines by site. Table outlining deployment of families and references lines.

Additional file 3: Table S3. Phenotype data. Data used for all analyses in manuscript.

Additional file 4: Table S4. Pedigree data. Pedigree information used for all analyses in manuscript.

Additional file 5: Table S5. Survival among reference lines. Table of survival of individual diploid and tetraploid reference lines.

Additional file 6: Table S6A and S6B. Fixed effects for survival. Tables of line and spawn effects for late spring and final survival.

Additional file 7: Table S7A and S7B. Variance components and heritabilities of survival and weight, double reduction = 0.074. Tables of variance components and heritabilities for survival and weight with double reduction = 0.074.

Additional file 8: Table S8A and S8B. Fixed effects for weight. Tables of line and spawn effects for spring weight and final weight.

## Data Availability

Phenotypic data and pedigree data used in this study are available as additional files.
