# Supplementary material for: Quantitative genetic analysis of late spring mortality in triploid Crassostrea virginica
Source: Genet Sel Evol. 2025 Apr 9;57:19. doi: 10.1186/s12711-025-00965-3 (PMC11983945; doi:10.1186/s12711-025-00965-3)
Supplement: Supplementary file 1 — Additional file 1: Table S1. Reference lines included in field trial. Table specifying reference lines in field trial. [file 12711_2025_965_MOESM1_ESM.docx]

**Table S1** **Reference lines included in field trial**

| Cross | Ploidy | Salinity |
| --- | --- | --- |
| LOLA | 2N | low |
| LILY | 2N | low |
| LFAMS | 2N | low |
| DEBY | 2N | high |
| XB | 2N | high |
| HNRY | 2N | high |
| 4GEN | 4N | – |
| 4GNL | 4N | – |
| 4VBOY | 4N | – |
| 4OBLT | 4N | – |

Diploid (2N) and tetraploid (4N) lines of *Crassostrea virginica* included in the field trial for reference to the survival of triploid and tetraploid families. Lines are distinguished by the salinity of the environment in which they have been selected: high (18-22 ppt) or low (8-12 ppt). Tetraploid lines have been reared in both the York River (Gloucester Point, Virginia; ≈ 18-22 ppt) and Rappahannock River (Topping, Virginia; ≈ 12-17 ppt).
