# Supplementary material for: Quantitative genetic analysis of late spring mortality in triploid Crassostrea virginica
Source: Genet Sel Evol. 2025 Apr 9;57:19. doi: 10.1186/s12711-025-00965-3 (PMC11983945; doi:10.1186/s12711-025-00965-3)
Supplement: Supplementary file 2 — Additional file 2: Table S2. Deployment of families and reference lines by site. Table outlining deployment of families and references lines. [file 12711_2025_965_MOESM2_ESM.docx]

**Table S2 Deployment of families and reference lines by site**

| Group | YR | CR | ND |  |
| --- | --- | --- | --- | --- |
| Triploid families | ✓ | ✓ | ✓ |  |
| Tetraploid families | ✓ | ✓ | – |  |
| Diploid high salinity reference lines | ✓ | – | ✓ |  |
| Diploid low salinity reference lines | – | ✓ | – |  |
| Tetraploid reference lines | ✓ | ✓ | ✓ |  |

Deployment of families and reference lines of *Crassostrea virginica* at York River (YR), Choptank River (CR), and Nandua Creek (ND) for the field test. Diploid high salinity reference lines refer to DEBY, XB, and HNRY, while low salinity reference lines refer to LOLA, LILY, and LFAMS. Tetraploid reference lines were 4GEN, 4GNL, 4VBOY, and 4OBLT. A “✓” signifies group was deployed, whereas “–” represents group not deployed.
