# Supplementary material for: Quantitative genetic analysis of late spring mortality in triploid Crassostrea virginica
Source: Genet Sel Evol. 2025 Apr 9;57:19. doi: 10.1186/s12711-025-00965-3 (PMC11983945; doi:10.1186/s12711-025-00965-3)
Supplement: Supplementary file 5 — Additional file 5: Table S5. Survival among reference lines. Table of survival of individual diploid and tetraploid reference lines. [file 12711_2025_965_MOESM5_ESM.docx]

**Table S5 Survival among reference lines**

| Line | YR | CR | ND |  | Salinity |
| --- | --- | --- | --- | --- | --- |
|  |  |  |  |  |  |
| *Diploids* | | | | | |
| LOLA | – | 0.50 (0.05) | – |  | low |
| LILY | – | 0.79 (0.04) | – |  | low |
| LFAMS | – | 0.74 (0.04) | – |  | low |
| DEBY | 0.72 (0.04) | – | 0.86 (0.05) |  | high |
| HNRY | 0.80 (0.05) | – | 0.90 (0.03) |  | high |
| XB | 0.82 (0.02) | – | 0.77 (0.08) |  | high |
|  |  |  |  |  |  |
| *Tetraploids* |  |  |  |  |  |
| 4GEN | 0.27 (0.05) | 0 | 0.48 (0.06) |  | – |
| 4GNL | 0.39 (0.05) | 0.09 (0.04) | 0.60 (0.07) |  | – |
| 4VBOY | * | 0 | 0.69 (0.02) |  | – |
| 4OBLT | 0.73 (0.02) | 0.24 (0.05) | 0.75 (0.03) |  | – |

Final survival of diploid and tetraploid reference lines of *Crassostrea virginica* at York River (YR), Choptank River (CR), and Nandua Creek (ND). Standard errors are in parentheses. “–” represents lines not deployed. Salinity refers to the environment in which each line has been selected: high (18-22 ppt) or low (8-12 ppt). *Survival of 4VBOY was not calculated at YR because of a data collection error.
