# Supplementary material for: Quantitative genetic analysis of late spring mortality in triploid Crassostrea virginica
Source: Genet Sel Evol. 2025 Apr 9;57:19. doi: 10.1186/s12711-025-00965-3 (PMC11983945; doi:10.1186/s12711-025-00965-3)
Supplement: Supplementary file 6 — Additional file 6: Table S6A and S6B. Fixed effects for survival. Tables of line and spawn effects for late spring and final survival. [file 12711_2025_965_MOESM6_ESM.docx]

|  | 4N_YR | 3N_YR | 3N_CR | 3N_ND |
| --- | --- | --- | --- | --- |
| Intercept | 0.86 (0.05) | 0.96 (0.01) | 0.86 (0.03) | 0.94 (0.04) |
| 4GEN | 0.00 | 0.00 | 0.00 | 0.00 |
| 4GNL | -0.07 (0.08) | 0.00 (0.02) | 0.06 (0.06) | -0.33 (0.08) |
| 4LGT | 0.13 (0.06) | 0.05 (0.02) | 0.14 (0.05) | 0.06 (0.07) |
| 4OBOY | 0.13 (0.07) | 0.03 (0.02) | 0.16 (0.05) | 0.02 (0.07) |
| 4VBOY | -0.10 (0.09) | 0.01 (0.03) | 0.14 (0.07) | -0.29 (0.09) |
| SPAWN 1 | 0.00 | – | – | – |
| SPAWN 2 | -0.02 (0.03) | – | – | – |
| DBY_LEW | – | 0.00 | 0.00 | 0.00 |
| DBY_LYN | – | -0.03 (0.03) | -0.05 (0.08) | -0.12 (0.13) |

**Table S6A** **Fixed effects for late spring survival**

Line and spawn effects from univariate models on late spring survival of triploid (3N) and tetraploid (4N) families of *Crassostrea virginica* measured at three sites in the Chesapeake Bay (York River, Choptank River, Nandua Creek). Standard errors are in parentheses. SPAWN 1 = June 14, 2017; SPAWN 2 = July 10, 2017.

**Table S6B Fixed effects for final survival**

|  | 4N YR | 3N YR | 3N CR | 3N ND |
| --- | --- | --- | --- | --- |
| Intercept | 0.53 (0.08) | 0.69 (0.03) | 0.37 (0.07) | 0.80 (0.06) |
| 4GEN | 0.00 | 0.00 | 0.00 | 0.00 |
| 4GNL | -0.17 (0.12) | 0.13 (0.06) | -0.01 (0.14) | -0.32 (0.13) |
| 4LGT | 0.19 (0.10) | 0.33 (0.05) | 0.46 (0.12) | 0.17 (0.11) |
| 4OBOY | 0.19 (0.11) | 0.21 (0.05) | 0.34 (0.12) | 0.10 (0.11) |
| 4VBOY | -0.11 (0.15) | 0.18 (0.07) | 0.29 (0.15) | -0.30 (0.14) |
| SPAWN_1 | 0.00 | – | – | – |
| SPAWN_2 | -0.10 (0.05) | – | – | – |
| DBY_LEW | – | 0.00 | 0.00 | 0.00 |
| DBY_LYN | – | -0.26 (0.08) | -0.21 (0.20) | -0.16 (0.20) |

Line and spawn effects from univariate models on final survival of triploid (3N) and tetraploid (4N) families of *Crassostrea virginica* measured at three sites in the Chesapeake Bay (York River, Choptank River, Nandua Creek). Standard errors are in parentheses. SPAWN 1 = June 14, 2017; SPAWN 2 = July 10, 2017.
