# Supplementary material for: Quantitative genetic analysis of late spring mortality in triploid Crassostrea virginica
Source: Genet Sel Evol. 2025 Apr 9;57:19. doi: 10.1186/s12711-025-00965-3 (PMC11983945; doi:10.1186/s12711-025-00965-3)
Supplement: Supplementary file 7 — Additional file 7: Table S7A and S7B. Variance components and heritabilities of survival and weight, double reduction = 0.074. Tables of variance components and heritabilities for survival and weight with double reduction = 0.074. [file 12711_2025_965_MOESM7_ESM.docx]

|  |  | 4N_YR | 3N_YR | 3N_CR | 3N_ND | | | |  |
| --- | --- | --- | --- | --- | --- | --- | --- | --- | --- |
| *Late spring survival (T2 to T3)* | | | | | |  | |  |  |
| $\text{σ}_{\text{a}}^{\text{2}}$ |  | 0.004 | < 0.001 | 0.003 | 0.013 | | | |  |
| $\text{σ}_{u}^{\text{2}}$ |  | 0.010 | 0.001 | 0.003 | 0.001 | | | |  |
| $\text{σ}_{\text{f}}^{\text{2}}$ |  | < 0.001 | – | – | – | | | |  |
| $\text{σ}_{\text{}}^{\text{2}}$ |  | 0.076 | 0.033 | 0.059 | 0.083 | | | |  |
| obs h^2^ |  | 0.05 (0.03) | 0.01 (0.01) | 0.05 (0.05) | 0.14 (0.06) | | | |  |
| und h^2^ |  | 0.13 (0.09) | 0.03 (0.07) | 0.12 (0.11) | 0.52 (0.21) | | | |  |
|  |  |  | | | | |  |  |  |
| *Final survival (T0 to T4)* | | | | | | |  |  |  |
| $\text{σ}_{\text{a}}^{\text{2}}$ |  | 0.017 | 0.001 | 0.024 | 0.031 | | | |  |
| $\text{σ}_{u}^{\text{2}}$ |  | 0.012 | 0.006 | 0.015 | 0.003 | | | |  |
| $\text{σ}_{\text{f}}^{\text{2}}$ |  | 0.003 | – | – | – | | | |  |
| $\text{σ}_{\text{}}^{\text{2}}$ |  | 0.206 | 0.170 | 0.197 | 0.130 | | | |  |
| obs h^2^ |  | 0.07 (0.05) | 0.003 (0.02) | 0.10 (0.06) | 0.19 (0.08) | | | |  |
| und h^2^ |  | 0.11 (0.08) | 0.01 (0.03) | 0.16 (0.11) | 0.39 (0.16) | | | |  |

**Table S7A Estimates of variance components and heritabilities of survival, double reduction = 0.074**

Estimates of additive genetic variation ($\text{σ}_{\text{a}}^{\text{2}}$), variation from unit effect ($\text{σ}_{u}^{\text{2}}$), variation from family effect ($\text{σ}_{\text{f}}^{\text{2}}$), residual variation ($\text{σ}_{\text{}}^{\text{2}}$), narrow-sense heritability (h^2^) on the observed (obs) and underlying scale (und) for late spring survival (survival between spring of 2019 and summer of 2019) and final survival (survival between summer of 2018 and fall of 2019) for triploid (3N) and tetraploid (4N) families of *Crassostrea virginica* measured at three sites in the Chesapeake Bay (York River, Choptank River, Nandua Creek). Pedigree set with double reduction frequency = 0.074. Standard errors are in parentheses. “–” indicates variable not in model. T0 = deployment in spring of 2018, T2 = spring of 2019, T3 = summer of 2019, T4 = fall of 2019.

**Table S7B Estimates of variance components and heritabilities of weight, double reduction = 0.074**

|  |  | 4N_YR | 3N_YR | 3N_CR | 3N_ND |  |
| --- | --- | --- | --- | --- | --- | --- |
| *Spring weight (T2)* | | | | | |  |
| $\text{σ}_{\text{a}}^{\text{2}}$ |  | 25.08 | 65.28 | 3.69 | < 0.001 |  |
| $\text{σ}_{u}^{\text{2}}$ |  | 14.06 | 5.03 | 4.92 | 18.16 |  |
| $\text{σ}_{\text{f}}^{\text{2}}$ |  | 0.71 | – | – | – |  |
| $\text{σ}_{r}^{\text{2}}$ |  | 58.28 | 45.26 | 50.47 | 118.62 |  |
| h^2^ |  | 0.26 (0.13) | 0.56 (0.20) | 0.06 (0.08) | < 0.001 (0.08) |  |
|  |  |  | | | | |
| *Final weight (T4)* | | | | | | |
| $\text{σ}_{\text{a}}^{\text{2}}$ |  | 28.95 | 243.72 | 104.89 | 40.46 |  |
| $\text{σ}_{u}^{\text{2}}$ |  | 34.06 | 16.80 | 35.42 | 17.89 |  |
| $\text{σ}_{\text{f}}^{\text{2}}$ |  | 40.79 | – | – | – |  |
| $\text{σ}_{r}^{\text{2}}$ |  | 237.59 | 284.46 | 147.34 | 313.57 |  |
| h^2^ |  | 0.08 (0.14) | 0.45 (0.17) | 0.36 (0.18) | 0.11 (0.08) |  |

Estimates of additive genetic variation ($\text{σ}_{\text{a}}^{\text{2}}$), variation from unit effect ($\text{σ}_{u}^{\text{2}}$), variation from family effect ($\text{σ}_{\text{f}}^{\text{2}}$), residual variation ($\text{σ}_{\text{}}^{\text{2}}$), and narrow-sense heritability (h^2^) for spring weight (weight in spring of 2019) and final weight (weight by fall of 2019) for triploid (3N) and tetraploid (4N) families of *Crassostrea virginica* measured at three sites in the Chesapeake Bay (York River, Choptank River, Nandua Creek). Pedigree set with double reduction frequency = 0.074. Standard errors are in parentheses. “–” indicates variable not in model. T2 = spring of 2019, T4 = fall of 2019.
