# Supplementary material for: Quantitative genetic analysis of late spring mortality in triploid Crassostrea virginica
Source: Genet Sel Evol. 2025 Apr 9;57:19. doi: 10.1186/s12711-025-00965-3 (PMC11983945; doi:10.1186/s12711-025-00965-3)
Supplement: Supplementary file 8 — Additional file 8: Table S8A and S8B. Fixed effects for weight. Tables of line and spawn effects for spring weight and final weight. [file 12711_2025_965_MOESM8_ESM.docx]

|  | 4N YR | 3N YR | 3N CR | 3N ND |
| --- | --- | --- | --- | --- |
| Intercept | 30.6 (2.7) | 50.6 (2.9) | 26.3 (1.1) | 43.2 (1.4) |
| 4GEN | 0.0 | 0.0 | 0.0 | 0.0 |
| 4GNL | 7.2 (4.3) | 8.5 (5.8) | 3.6 (2.4) | 6.3 (3.3) |
| 4LGT | 8.7 (3.5) | 3.4 (5.1) | 8.4 (2.0) | 8.6 (2.8) |
| 4OBOY | 16.2 (3.7) | 3.0 (5.0) | 8.5 (2.0) | 16.0 (2.8) |
| 4VBOY | 16.9 (5.3) | 2.5 (6.6) | 0.6 (2.6) | 5.6 (3.7) |
| SPAWN_1 | 0.0 | – | – | – |
| SPAWN_2 | -2.0 (1.9) | – | – | – |
| DBY_LEW | – | 0.0 | 0.0 | 0.0 |
| DBY_LYN | – | -32.1 (9.1) | -12.55 (3.2) | 3.0 (4.1) |

**Table S8A** **Fixed effects for spring weight**

Line and spawn effects from univariate models on spring weight for triploid (3N) and tetraploid (4N) families of *Crassostrea virginica* measured at three sites in the Chesapeake Bay (York River, Choptank River, Nandua Creek). Standard errors are in parentheses. SPAWN 1 = June 14, 2017; SPAWN 2 = July 10, 2017.

**Table S8B** **Fixed effects for final weight**

|  | 4N YR | 3N YR | 3N CR | 3N ND |
| --- | --- | --- | --- | --- |
| Intercept | 59.4 (5.2) | 118.5 (5.7) | 63.0 (4.1) | 78.6 (2.8) |
| 4GEN | 0.0 | 0.0 | 0.0 | 0.0 |
| 4GNL | 6.8 (8.4) | 21.7 (11.4) | 9.6 (8.4) | 14.7 (5.9) |
| 4LGT | 22.5 (6.7) | 18.2 (9.9) | 20.2 (7.3) | 10.8 (5.1) |
| 4OBOY | 33.0 (7.2) | 15.2 (9.7) | 23.6 (7.1) | 33.1 (5.1) |
| 4VBOY | 22.8 (9.9) | 4.9 (12.9) | -0.7 (9.4) | -0.4 (6.7) |
| SPAWN_1 | 0.0 | – | – | – |
| SPAWN_2 | -4.0 (3.3) | – | – | – |
| DBY_LEW | – | 0.0 | 0.0 | 0.0 |
| DBY_LYN | – | -48.8 (17.9) | -30.5 (12.7) | -0.3 (8.6) |

Line and spawn effects from univariate models on final weight for triploid (3N) and tetraploid (4N) families of *Crassostrea virginica* measured at three sites in the Chesapeake Bay (York River, Choptank River, Nandua Creek). Standard errors are in parentheses. SPAWN 1 = June 14, 2017; SPAWN 2 = July 10, 2017.
